# A Review of the Evidence to Support Influenza Vaccine Introduction in Countries and Areas of WHO's Western Pacific Region

**DOI:** 10.1371/journal.pone.0070003

**Published:** 2013-07-16

**Authors:** Gina Samaan, Michelle McPherson, Jeffrey Partridge

**Affiliations:** DSE, World Health Organization, Western Pacific Regional Office, Manila, Philippines; Fudan University, China

## Abstract

**Background:**

Immunization against influenza is considered an essential public health intervention to control both seasonal epidemics and pandemic influenza. According to the World Health Organization (WHO), there are five key policy and three key programmatic issues that decision-makers should consider before introducing a vaccine. These are (a) public health priority, (b) disease burden, (c) efficacy, quality and safety of the vaccine, (d) other inventions, (e) economic and financial issues, (f) vaccine presentation, (g) supply availability and (h) programmatic strength. We analyzed the body of evidence currently available on these eight issues in the WHO Western Pacific Region.

**Methodology/Principal Findings:**

Studies indexed in PubMed and published in English between 1 January 2000 and 31 December 2010 from the 37 countries and areas of the Western Pacific Region were screened for keywords pertaining to the five policy and three programmatic issues. Studies were grouped according to country income level and vaccine target group. There were 133 articles that met the selection criteria, with most (90%) coming from high-income countries. Disease burden (n = 34), vaccine efficacy, quality and safety (n = 27) and public health priority (n = 27) were most frequently addressed by studies conducted in the Region. Many studies assessed influenza vaccine policy and programmatic issues in the general population (42%), in the elderly (24%) and in children (17%). Few studies (2%) addressed the eight issues relating to pregnant women.

**Conclusions/Significance:**

The evidence for vaccine introduction in countries and areas in this Region remains limited, particularly in low- and middle-income countries that do not currently have influenza vaccination programmes. Surveillance activities and specialized studies can be used to assess the eight issues including disease burden among vaccine target groups and the cost-effectiveness of influenza vaccine. Multi-country studies should be considered to maximize resource utilization for cross-cutting issues such as vaccine presentation and other inventions.

## Introduction

The Western Pacific Region of the World Health Organization (WHO) comprises 37 countries and areas, spanning from China in the north and west, to New Zealand in the south, and to French Polynesia in the east [1]. One of the most diverse regions of WHO, the Western Pacific Region is home to approximately 1.6 billion people and includes highly developed countries as well as countries with rapidly emerging economies.

Awareness of the public health importance of influenza has increased in this Region in recent years, motivated by the emergence of highly pathogenic avian influenza A(H5N1) and subsequently by the occurrence of the A(H1N1)pdm09 pandemic. The Region currently has 21 National Influenza Centres (NICs) in 15 countries that monitor the impact and evolution of influenza viruses and inform global vaccine strain selection [2]. Influenza vaccination policies are established in 18 countries and areas, with another seven providing vaccine recommendations (unpublished data).

**Figure 1:**
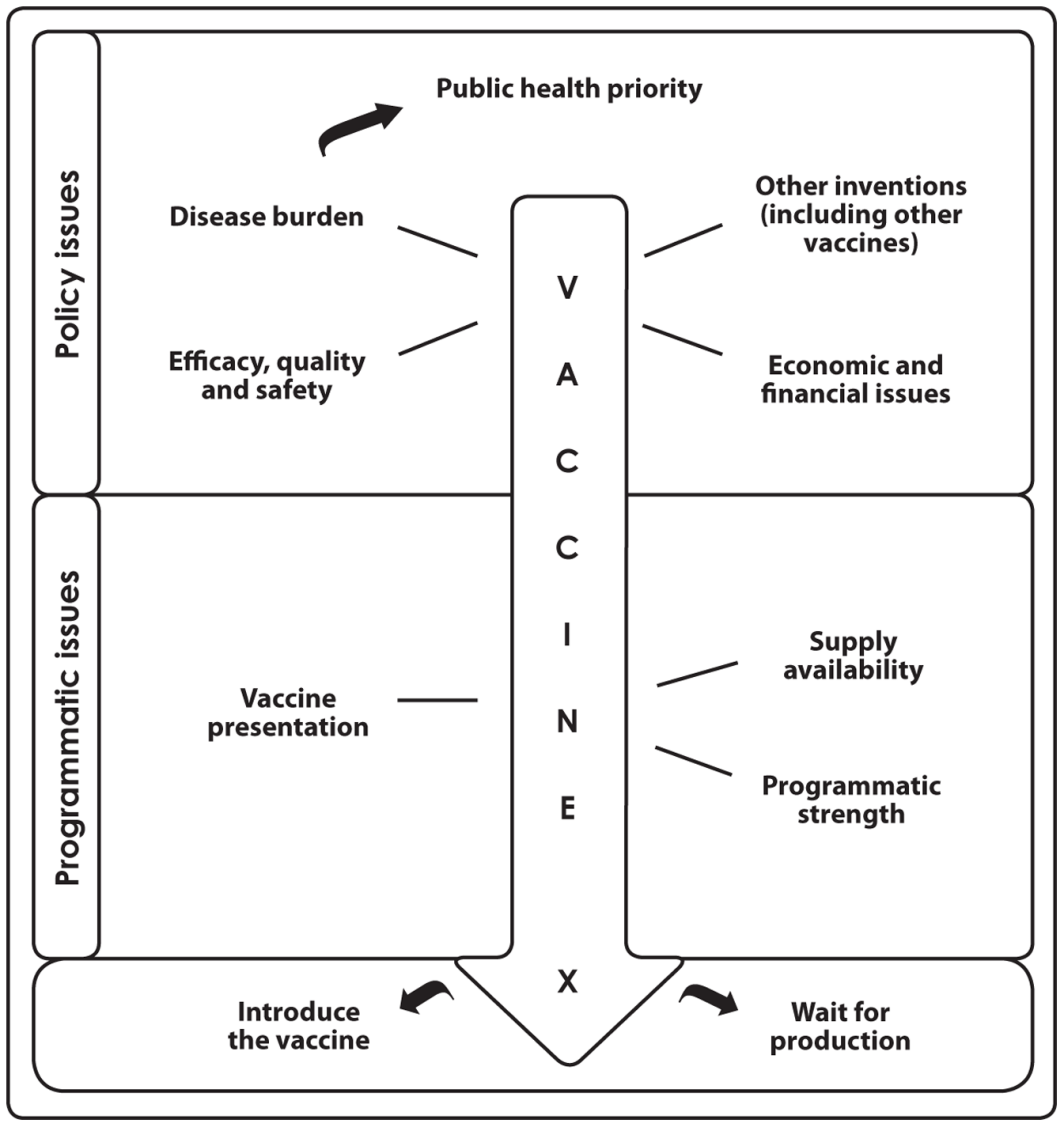
Flowchart of key issues to be considered before vaccine introduction [Source: WHO, 3].

To assist countries with the development of vaccine policy, WHO published the *Vaccine Introduction Guidelines* in 2005 [3]. These guidelines highlight five key policy issues and three key programmatic issues that decision-makers should consider before introducing a vaccine (Figure 1). In 2012, WHO published new recommendations for the use of influenza vaccines [4]. WHO recommends that pregnant women should have the highest priority for influenza vaccination. Additional risk groups to be considered for vaccination, in no particular order of priority, are children aged 6–59 months, the elderly, individuals with specific chronic medical conditions, and health care workers.

To better inform policy on influenza vaccine introduction in the Region, a literature review was conducted to summarize the body of evidence currently available on the eight policy and programmatic issues outlined in the WHO guidelines and in the context of the new recommendations for use of influenza vaccines. Although vaccine policy is developed and established by each country, reference to evidence from across the Region can support decision-making and identify future research needs that may be addressed collectively.

## Materials and Methods

**Figure 2:**
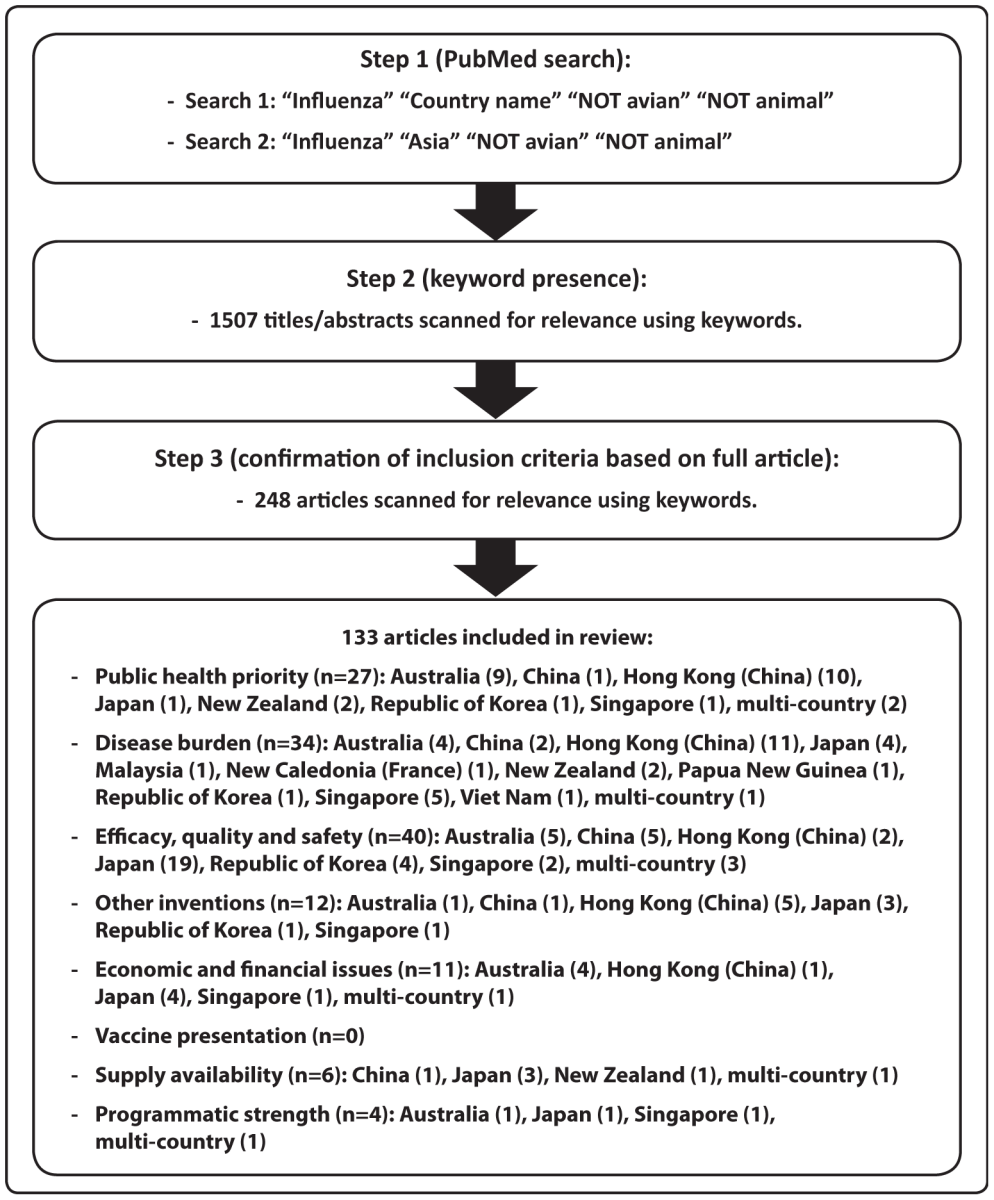
Methods to identify studies included in the literature review.

Data for this review were identified through a PubMed search and references from relevant articles. PubMed was used as it includes more than 19 million citations to biomedical literature from MEDLINE and life science journals, and it enabled the use of medical subject headings (MeSH) terms that are useful to explore publications based on key designated terms. Studies published in English between 1 January 2000 and 31 December 2010 from any of the 37 countries or areas of the Western Pacific Region [1] were included. The titles and abstracts of articles that included the search terms were screened for keywords pertaining to the five policy issues and three programmatic issues as per the WHO *Vaccine Introduction Guidelines* (Table 1), and the full articles that contained the keywords were collected and reviewed to confirm that the inclusion criteria were met (Figure 2).

**Table 1:** Keywords used to include studies in the literature review and to allocate studies to the relevant issue according to the WHO *Vaccination Introduction Guidelines*.

Key issues as per the WHO *Vaccine Introduction Guidelines*	Keywords used to classify studies to these issues
Public health priority	Priority, perception, Millennium Development Goals
Disease burden	Burden, incidence, prevalence, hospitalization, impact, mortality, cost, deaths, epidemiology, characteristic, etiology
Efficacy, quality and safety	Vaccine, efficacy, quality, safety, effectiveness, adverse event, standards, clinical trial
Other inventions	Antiviral, non-pharmaceutical
Economic and financial issues	Economic, budget, finance, funding, sustainability, cost-effectiveness, affordability, fiscal impact
Vaccine presentation	Presentation, formulation, dose
Supply availability	Supply, availability, manufacture, procurement, introduction strategy
Programmatic strength	Delivery, National Immunization Programme
Key issues as per the WHO Vaccine Introduction Guidelines	Keywords used to classify studies to these issues
Public health priority	Priority, perception, Millennium Development Goals
Disease burden	Burden, incidence, prevalence, hospitalization, impact, mortality, cost, deaths, epidemiology, characteristic, etiology
Efficacy, quality and safety	Vaccine, efficacy, quality, safety, effectiveness, adverse event, standards, clinical trial
Other inventions	Antiviral, non-pharmaceutical
Economic and financial issues	Economic, budget, finance, funding, sustainability, cost-effectiveness, affordability, fiscal impact
Vaccine presentation	Presentation, formulation, dose
Supply availability	Supply, availability, manufacture, procurement, introduction strategy
Programmatic strength	Delivery, National Immunization Programme

Studies were excluded if they (a) stated virus name but focused on other diseases, (b) stated virus name that incorporated the countries of interest but did not involve research in any Western Pacific Region country or area, or (c) were a publication of non-original research data such as outbreak news reports, editorials and reviews. Studies included in the final analysis were categorized, summarized and appraised according to the relevant key policy or programmatic issues [3] and were reported by the income level of the country or area (high versus low and middle income as based on the World Bank classifications [5]) and by the five target groups recommended by the WHO position paper for influenza vaccination as well as studies that focus on the general population [4].

## Results

The PubMed search using designated terms returned 1507 articles, of which 133 met the selection criteria and were categorized according to the WHO *Vaccine Introduction Guidelines* key issues (Figure 2). These studies were from 11 countries or areas (Australia, China, Hong Kong [China], Japan, Malaysia, New Caledonia [France], New Zealand, Papua New Guinea, the Republic of Korea, Singapore and Viet Nam).

### Public health priority

Public health priority comprises issues associated with prioritizing a particular vaccine over other competing public health issues. Twenty-seven studies focused on the prioritization of influenza vaccination within various target groups, of which 96% (n = 26) were from high-income countries (Table 2). Eight studies focused on health care workers; three assessed perceptions and vaccine uptake among health care workers [6]–[8], four on the need for further education for health care workers to increase their vaccine coverage rates [9]–[12] and one on their role in impacting the likelihood of influenza vaccination for the elderly [13]. Nine other studies assessed the factors that impact the likelihood of vaccination among the elderly [13]–[17], individuals with chronic conditions [18], [19] and pregnant women [20], [21].

**Table 2:** Number of studies conducted for the eight key issues, by country income classification and vaccination target groups, N = 133*.

Key Issue (Number of studies)	Number of studies by country income		Number of studies by target group					
	High income	Low and middle income	Children under 5 years	Health care workers	Pregnant women	People with chronic conditions/who are immunocompromised	Elderly	General population
Public health priority (N = 27)	26	1	0	9	2	3	10	4
Disease burden (N = 34)								
(a) Incidence or prevalence (n = 12)	10	2	3	0	0	0	2	6
(b) Hospitalization (n = 15)	13	2	5	0	0	1	1	6
(c) Mortality (n = 9)	7	2	1	0	0	0	1	7
Efficacy, quality and safety (N = 39)								
(a) Efficacy (n = 27)	22	5	9	3	0	1	4	11
(b) Effectiveness (n = 10)	10	0	2	0	0	2	4	0
(c) Safety (n = 9)	7	2	2	2	0	0	2	4
Other inventions (N = 12)	11	1	0	0	0	0	0	12
Economic and financial issues (N = 11)	11	0	0	0	0	0	7	4
Vaccine presentation (N = 0)	0	0	0	0	0	0	0	0
Supply availability (N = 6)	5	1	0	0	1	1	0	0
Programmatic strength (N = 4)	4	0	1	2	0	0	1	2

*Some studies report on more than one key issue or target group.

Research also assessed the normative beliefs that favour vaccination, the underlying health belief models and strategies for appealing to these beliefs, and the increasing accessibility to immunization in the community – among the general public [22], [23] and the elderly [24]–[26]. Five other studies focused on concerns held by the public [27] and risk groups such as the elderly [28], [29], health care workers [30] and cancer patients over vaccine safety, efficacy and development [31]. Lastly, one multi-country study assessed how influenza is perceived by the general population and which populations are perceived as appropriate vaccination targets [32].

### Disease burden

Thirty-four studies described disease burden, which was expressed in terms of (a) incidence or prevalence, (b) hospitalization and (c) mortality. Only 15% (n = 5) of studies were from low- and middle-income countries (Table 2). Since disease burden forms the basis for vaccine-introduction policy, each of the studies identified through the literature review has been summarized and tabulated (see Tables S1, S2, S3).

#### (a) Incidence or prevalence

Incidence or prevalence of influenza was assessed in 12 studies (83% [n = 10] from high-income countries or areas), with a focus on the general population [33]–[38] and risk groups such as indigenous people [39], children [37], [40], [41], the elderly [36], [38] and military populations [42]. The relationship between the incidence of influenza and climate parameters such as rainfall and humidity was also explored for a number of cities in the Region [43]. One study focused on methods for enumerating disease incidence [44].

#### (b) Hospitalization

Fifteen studies assessed influenza-associated hospitalization (87% [n = 13] from high-income countries or areas), with most studies focusing on describing rates in the general population [45]–[50] and risk groups such as the elderly [48], individuals with chronic conditions [51], children [52]–[56] and indigenous populations [54], [57]. Two studies from Hong Kong (China) compared influenza hospitalization rates to those in temperate regions [50], [52]. One study assessed admissions to intensive care units [58], and another assessed the direct costs of hospitalization for influenza patients during the 2009 pandemic [59].

#### (c) Mortality

Nine studies assessed influenza-associated mortality (seven from high-income countries or areas) by measuring excess mortality due to seasonal influenza [50], [60]–[64] and novel pandemic influenza in the general population [46], comparing mortality rates to findings in temperate countries [50], [61], [63], or assessing the impact of influenza vaccination on child mortality [65] or elderly mortality [66].

### Efficacy, quality and safety

Efficacy, quality and safety were addressed by 39 studies in three areas: (a) vaccine efficacy to ensure the vaccine prevents the disease in the immunized population; (b) vaccine effectiveness to ensure that protection is achieved under programmatic implementation in the target population; and (c) vaccine safety to ensure that the safety profile is well-understood and that the vaccine meets international standards of quality and safety.

#### (a) Efficacy

Twenty-seven studies assessed efficacy or immunogenicity of influenza vaccines, of which 19% (n = 5) were from low- and middle-income countries and 11% (n = 3) were multi-country studies (Table 2). For seasonal influenza, three studies assessed vaccine efficacy in the general population [67]–[69], eight studies in children [68], [70]–[76], four studies in elderly populations [68], [77]–[79], two in health care workers [80], [81] and one in immunocompromised haemodialysis patients [82].

Nine additional studies assessed the immunogenicity of A(H1N1)pdm09 vaccines in the general population [83]–[89], in children [90] and in health care workers [91]. These studies were conducted in Australia, China, Hong Kong (China), Japan, the Republic of Korea and Singapore. One study conducted in Hong Kong (China) assessed the immunogenicity of pre-pandemic H5N1 vaccines in the general population [92]. Lastly, a qualitative study reported on the dangers of intentional misinterpretation of vaccine efficacy data by anti-vaccination campaigners, including the impact on vaccination rates and doctors' willingness to recommend vaccination [93].

#### (b) Effectiveness

Ten studies, all conducted in high-income countries and 80% (n = 8) in Japan, assessed influenza vaccine effectiveness (Table 2). Studies evaluated vaccine effectiveness in children [94], [95], the elderly [96]–[99] and the immunocompromised [95], [100]. Three studies reported on methods for estimating vaccine effectiveness including using routine surveillance data [101], school-based rapid diagnostic testing [102] and online surveys [103].

#### (c) Safety

Nine studies assessed influenza vaccine safety, of which only 22% (n = 2) were from low- and middle-income countries (Table 2). Four studies evaluated adverse events associated with seasonal influenza vaccines; one was conducted in children [76], one in health care workers [104], one in the general population [68] and two in the elderly [68], [77]. Pandemic A(H1N1)pdm09 vaccines were assessed for their safety in the general population [83], [105], in health care workers [106] and in children [90]. The pre-pandemic AS03-adjuvanted H5N1 vaccine was assessed for its safety profile in one study in the general population [92].

### Other inventions

Twelve studies on other inventions were conducted in the Region, of which only 8% (n = 1) was from a low- and middle-income country (Table 2). All the studies were conducted in the general population. Research was conducted on hand hygiene [107], [108], infection control [109], facemask use [108], [110], school closure [111], [112], physical exercise [113], alternative therapies including tea catechin [114], passive immunotherapy for management of severe cases of influenza infection [115] and use of traditional medicine [116]. One modelling study assessed methods to improve the effectiveness of different antiviral strategies [117], and another evaluated combinations of methods including enhanced surveillance with isolation, segregation and personal protective equipment to limit influenza transmission in closed environments [118].

### Economic and financial issues

Eleven studies, all from high-income countries, assessed the cost-effectiveness or economic efficiency for seasonal and pre-pandemic influenza vaccine (Table 2). Seven studies focused on the elderly, of which four evaluated the cost-effectiveness of seasonal influenza vaccination for the elderly [119]–[122], two assessed the cost-effectiveness of combining influenza and pneumococcal vaccination compared to influenza vaccination alone [120], [123], and two assessed the impact of subsidizing the cost of vaccination [124], [125]. One study from Hong Kong (China) assessed the cost-effectiveness of vaccine including subsidy in the general population [126]. One multi-country study assessed vaccine coverage in relation to gross national income per capita as well as the impact of increasing income and education on coverage rates [32]. Two studies assessed the factors that impact cost-effectiveness of pre-pandemic and pandemic vaccine including vaccine strain match, availability and cost [127], [128].

### Vaccine presentation

No studies met the criteria for vaccine presentation, formulation or dosage.

### Supply availability

Six studies examined influenza vaccine supply and production issues (Table 2). The five studies conducted by individual countries reviewed national vaccine needs and production capacities [129], [130] including for pregnant women [131] and people with chronic conditions [131] (Table 2), discussed challenges of having limited suppliers for seasonal influenza vaccine and the potential implications of supply disruption [132], and presented plans for safety tests for pre-pandemic candidate vaccines to inform vaccine introduction policy [133]. A multi-country study assessed global vaccine usage and an increase in uptake between 1994 and 2003 [134].

### Programmatic strength

Four studies, all from high-income countries, assessed programmatic strengths to deliver influenza vaccine to the target populations (Table 2). Two studies assessed methods to improve the uptake of influenza vaccination by hospital-based health care workers, including conducting onsite vaccination clinics [135] and providing senior management support for vaccination [136]. One study described lessons learnt for increasing vaccination rates among high-risk groups, including likely hindrances such as the vocal anti-vaccination campaign and the reporting of improperly conducted vaccine efficacy studies [137]. The fourth study was a multi-country study that assessed influenza vaccination coverage rates among adults, the elderly and children [32].

## Discussion

Immunization against influenza is considered an essential public health intervention to control both seasonal epidemics and pandemic influenza [138]. In the Western Pacific Region, published literature on influenza vaccine policy and programmatic issues is mostly limited to countries with existing influenza vaccination programmes. Of the 11 countries and areas with studies included in this report, eight reported that seasonal influenza vaccine was available through both government funding and private market purchase, two reported vaccine was available through private market purchase only (Singapore and Viet Nam) and one reported the lack of any vaccination programme (Papua New Guinea, unpublished data).

Most research conducted in the Region focused on the general population and the elderly. Future work should consider other target groups recommended for influenza vaccine by WHO, especially pregnant women since this group is deemed a priority [4].

The perception of the public and the medical community on influenza disease and vaccines is a significant factor in determining if vaccine introduction is a priority [3]. No research was found that linked influenza to national health priorities, Millennium Development Goals or national decision-making groups that may be relevant to countries considering vaccine-introduction policy.

Defining disease burden is key to providing the rationale for vaccine introduction. However, although 34 disease burden studies were identified, the majority were conducted in countries and areas that have already established influenza vaccination policy (Australia, Hong Kong [China], Japan, New Zealand and Singapore). As many countries in the Region have not yet published data on influenza disease burden, future work is needed to understand the burden in different populations and different risk groups. Most studies analysed data arising from influenza surveillance systems that linked case counts to laboratory findings and hospitalization and mortality registries. Many countries in the Region have sentinel surveillance systems for influenza, which can be used to inform disease burden. Surveillance system data not only inform the decision to introduce vaccine, but also enable evaluation and continued measurement of vaccine impact. Options for determining disease burden include (a) utilizing data from countries of similar social and demographic characteristics, (b) deriving estimates from mathematical modelling and (c) conducting active surveillance studies. Specialized and targeted studies would be most useful in countries with limited laboratory access [3].

Research on vaccine efficacy, effectiveness and safety in the Region adds evidence that influenza vaccines are safe and provide adequate protection when matched to circulating strains. The body of evidence provided in these areas, which included both seasonal and pandemic influenza vaccination, was mainly from high-income countries, with Japan conducting the majority of studies on vaccine effectiveness. Studies on adverse events from influenza vaccination have been conducted in three of the target groups for vaccination, namely children, health care workers and the elderly. Despite the evidence presented, studies from more countries in the Region are needed to determine the efficacy, effectiveness and safety of influenza vaccines in different populations and settings and to assess their suitability in different countries across the Region. Since research in this area is resource-intensive, enhanced cross-country collaboration and public–private partnerships, as well as ongoing participation in the Global Influenza Surveillance and Response System (GISRS), are recommended [139].

Some studies focused on other public health measures as alternatives to vaccination. The effectiveness of these other mechanisms should be determined and shared to support the exploration of such public health measures being considered in other regions.

The body of published literature on the economic and financial aspects of influenza vaccine policy is very limited. None of the countries in the Region currently without influenza vaccination policy conducted research on the potential costs and benefits of adding influenza vaccine to their schedule or the potential impact it would have on limited national health budgets. Assessing the economic and financial implications of new vaccines should be considered carefully so that decision-makers can assess (a) the cost-effectiveness relative to other uses of scare resources, (b) the long-term resource requirements, (c) the funding gaps and whether additional domestic or external funding could be mobilized, and (d) the potential financial sustainability of the new vaccine [3].

A Global Action Plan was developed to increase seasonal vaccine use, increase production capacity and enable research and development [140]. There is evidence that vaccine production capacity is increasing globally [138]. In 2009, countries of the Western Pacific Region were projected to produce 23% (133 million doses) of the 573 million doses of seasonal trivalent vaccine produced globally [138]. Yet, as influenza vaccine production increases globally and more countries are considering introducing influenza vaccine policy [134], [138], [140], gaps in knowledge remain about country-level preferences for vaccine formulations and dosing, vaccination delivery systems, the additional resources required and the marketing component required to promote vaccine uptake.

This literature review had some limitations. First, only studies published in English were considered, which may have excluded a large volume of research in national languages, especially research that is nationally relevant but of limited international interest, such as programmatic issues. Second, the literature review searched only for studies indexed by PubMed, which may have underestimated the volume of research and excluded research on socio-behavioural or operational research, which are more likely to be indexed by other databases or in “grey” literature [141]. Nevertheless, PubMed is of value as it reflects the knowledge that is widely available for shared learning nationally and internationally.

In conclusion, the evidence for vaccine introduction in countries and areas in the Western Pacific Region remains limited, particularly in low- and middle-income countries that do not currently have influenza vaccination programmes. Few countries have conducted analyses of disease burden that provide the basis for vaccine introduction policy. To move forward, countries with influenza surveillance systems, especially those with National Influenza Centres carrying out virological surveillance, may consider utilizing surveillance activities and specialized studies to assess key issues, including identification of risk groups, seasonal trends and costs of influenza, and the cost-effectiveness of influenza vaccine. Importantly, a number of multi-country studies were conducted in the Region over the past 10 years, and opportunities for further collaboration should be explored to maximize resource utilization.

## Supporting Information

Table S1 **Studies reporting on influenza incidence and prevalence.**
(DOCX)

Table S2 **Studies reporting on influenza-associated hospitalization.**
(DOCX)

Table S3 **Studies reporting on influenza-associated mortality.**
(DOCX)
