# Supplementary material for: A Review of the Evidence to Support Influenza Vaccine Introduction in Countries and Areas of WHO's Western Pacific Region
Source: PLoS One. 2013 Jul 16;8(7):e70003. doi: 10.1371/journal.pone.0070003 (PMC3713047; doi:10.1371/journal.pone.0070003)
Supplement: Table S1 — Studies reporting on influenza incidence and prevalence. (DOCX) [file pone.0070003.s001.docx]

Supplementary Table 1: Studies reporting on influenza incidence and prevalence

| **Author** | **Country** | **Year(s)** | **Study description** | **Age group** | **Key findings** |
| --- | --- | --- | --- | --- | --- |
| Nelson et al [[1](#_ENREF_1)] | Hong Kong SAR(China) | 1997-1999 | Retrospective study of hospital discharge data from 1 hospital | <15 years | Incidence rates of respiratory illness coded as bronchiolitis and influenza were respectively estimated to be 887-979 and 222-381 per 100,000 children <5 years, and 3551-3949 and 415-528 per 100,000 children <1 year old. |
| Puzelli et al [[2](#_ENREF_2)] | Papua New Guinea | 2001-2002 | Cohort study involving 47 villages with 11,504 population in highlands | ≥15 years | Both influenza A (subtypes H3N2 and H1N1) and influenza B circulated in villages with higher prevalence rates in peripheral villages compared to those closer to the district headquarter. Determinants for variation in seroprevalence were not explored. |
| Leo et al [[3](#_ENREF_3)] | Singapore | 2009 | Cohort study in one hospital | All | In the first month of 2009 pandemic, seasonal influenza, predominantly virus subtype H3N2, was diagnosed for 32% of patients with acute febrile respiratory illness. |
| Phuong et al [[4](#_ENREF_4)] | Viet Nam | 2001-2005 | 606 patients in 12 healthcare centers in south Viet Nam | All | Paired sera showed 2.2% were influenza B virus and 1.9% influenza A virus infections. Some inter-annual differences were observed but no association with age or sex. Antibody prevalence, indicative of previous infections, was relatively low: 12.1% for influenza B and 5.9% influenza A viruses. |
| Seah et al [[5](#_ENREF_5)] | Singapore | 2006-2007 | 1,354 military recruits reporting ILI | >16 years | Influenza A virus was the dominant infection 24% (326/1354), with H3N2 subtype predominance. The temporal pattern coincided with the pattern in the civilian community. Influenza vaccination was recommended for this sub-population. |
| Flint et al [[6](#_ENREF_6)] | Australia | June-August 2009 | Retrospective analysis of influenza notifications and hospital data | All | Adjusted incidence rate ratio of pandemic virus was greater for indigenous population (269 per 100,000) compared to non-indigenous population (29 per 100,000). |
| Wada et al [[7](#_ENREF_7)] | Japan | 1998-2002 | 472 cases of influenza-related encephalopathy | ≤15 years | Compared to patients 0-5 years, patients 6-15 years had a significantly greater incidence of type B infection, lower frequency of convulsions, higher frequency of loss of consciousness and altered consciousness as the initial neurological symptom, lower serum transaminase levels, lower frequency of low-density area for brain CT* upon admission, and lower incidence of sequelae. |
| Huang et al [[8](#_ENREF_8)] | New Zealand | 1997-2006 | Retrospective population based study using data from sentinel general practice surveillance, laboratory-based surveillance, hospital admission, mortality surveillance and immunisation coverage | All | Sentinel surveillance showed that children aged 0-4 years were the most affected. Influenza-related hospitalisation surveillance reported an increasing trend of hospital admissions particularly in children aged 0-19 years. Introduction of routine influenza vaccination among elderly was associated with a significant decrease of influenza-related mortality. |
| Lau et al [[9](#_ENREF_9)] | Hong Kong SAR(China) | 1998-2007 | Retrospective population based study using data from multiple influenza-related surveillance systems | All | Use of multiple streams of influenza surveillance data can improve the accuracy and timeliness of alerts compared with monitoring of aggregate data or of any single stream alone. |
| Ng et al [[10](#_ENREF_10)] | Singapore | 2001 | Retrospective study using community sample surveys, national virological surveillance, hospitalization and mortality data. | All | An estimated 630,000 cases of influenza virus infection occur yearly, leading to 520,000 sick visits and 315,000 days of sick absence from work. About 4200 elderly (65+) persons were hospitalised for pneumonia and influenza, resulting in about 1,450 deaths every year. With vaccination, up to 315,000 cases, 258,000 sick visits, and 157,000 lost days from work would be prevented yearly, along with 2,100 hospitalisations and 600 deaths from pneumonia in the elderly. |
| Tang et al [[11](#_ENREF_11)] | Hong Kong SAR(China) | 2000-2007 | Retrospective study of respiratory virus incidence data from four hospitals and climatic data from Hong Kong Observatory | <18 years | Influenza A and RSV incidence increased with higher environmental relative humidity, whereas influenza B incidence decreased with higher environmental temperatures. Climiate factors and respiratory virus incidence differ between subtropical/tropical and temperate countries. |
| Tang et al [[12](#_ENREF_12)] | Multi-country | 2000-2007 | Time-series analysis | - | Relative humidity was associated with the incidence of influenza A in Singapore, Hong Kong and Brisbane.  Mean temperature was associated with the incidence of influenza B in Hong Kong, Brisbane, Melbourne, and Vancouver. |

* CT – computed tomography

References

1. Nelson EA, Tam JS, Yu LM, Li AM, Chan PK, et al. (2007) Assessing disease burden of respiratory disorders in Hong Kong children with hospital discharge data and linked laboratory data. Hong Kong Med J 13: 114-121.

2. Puzelli S, Boros S, Affinito C, Calzoletti L, Facchini M, et al. (2006) Prevalence of antibodies against A and B influenza viruses in South-Western Papua New Guinea. J Med Virol 78: 820-824.

3. Leo YS, Lye DC, Barkham T, Krishnan P, Seow E, et al. (2010) Pandemic (H1N1) 2009 surveillance and prevalence of seasonal influenza, Singapore. Emerg Infect Dis 16: 103-105.

4. Phuong HL, Nga TT, van Doornum GJ, Groen J, Binh TQ, et al. (2010) Viral respiratory tract infections among patients with acute undifferentiated fever in Vietnam. Southeast Asian J Trop Med Public Health 41: 1116-1126.

5. Seah SG, Lim EA, Kok-Yong S, Liaw JC, Lee V, et al. (2010) Viral agents responsible for febrile respiratory illnesses among military recruits training in tropical Singapore. J Clin Virol 47: 289-292.

6. Flint SM, Davis JS, Su JY, Oliver-Landry EP, Rogers BA, et al. (2010) Disproportionate impact of pandemic (H1N1) 2009 influenza on Indigenous people in the Top End of Australia's Northern Territory. Med J Aust 192: 617-622.

7. Wada T, Morishima T, Okumura A, Tashiro M, Hosoya M, et al. (2009) Differences in clinical manifestations of influenza-associated encephalopathy by age. Microbiol Immunol 53: 83-88.

8. Huang QS, Lopez LD, McCallum L, Adlam B (2008) Influenza surveillance and immunisation in New Zealand, 1997-2006. Influenza Other Respi Viruses 2: 139-145.

9. Lau EH, Cowling BJ, Ho LM, Leung GM (2008) Optimizing use of multistream influenza sentinel surveillance data. Emerg Infect Dis 14: 1154-1157.

10. Ng TP, Pwee KH, Niti M, Goh LG (2002) Influenza in Singapore: assessing the burden of illness in the community. Ann Acad Med Singapore 31: 182-188.

11. Tang JW, Lai FY, Wong F, Hon KL (2010) Incidence of common respiratory viral infections related to climate factors in hospitalized children in Hong Kong. Epidemiol Infect 138: 226-235.

12. Tang JW, Lai FY, Nymadawa P, Deng YM, Ratnamohan M, et al. (2010) Comparison of the incidence of influenza in relation to climate factors during 2000-2007 in five countries. J Med Virol 82: 1958-1965.
