# Supplementary material for: A Review of the Evidence to Support Influenza Vaccine Introduction in Countries and Areas of WHO's Western Pacific Region
Source: PLoS One. 2013 Jul 16;8(7):e70003. doi: 10.1371/journal.pone.0070003 (PMC3713047; doi:10.1371/journal.pone.0070003)
Supplement: Table S2 — Studies reporting on influenza-associated hospitalization. (DOCX) [file pone.0070003.s002.docx]

Supplementary Table 2: Studies reporting on influenza-associated hospitalization

| **Author** | **Country** | **Year(s)** | **Study description** | **Age group** | **Key findings** |
| --- | --- | --- | --- | --- | --- |
| Verrall et al [[1](#_ENREF_1)] | New Zealand | 2009 | Retrospective review of hospitalization data from hospitals in two regions | All | Pacific and Maori population 5-7 times more likely than European New Zealanders to require hospitalization for A(H1N1)pdm09   - European: 25.6 per 100,000 - Pacific Islanders: 180 per 100,000 - Maori: 128 per 100,000 |
| Yu et al [[2](#_ENREF_2)] | China | 2009 | Monte Carlo simulation model | All | Assuming 1968 pandemic strain severity, a pandemic would lead to 1.94-2.27 million hospitalizations |
| Li et al [[3](#_ENREF_3)] | Hong Kong SAR(China) | 1999-2000 | Retrospective review of hospitalization data from patient records | All | Mean excess numbers of hospitalizations attributable to influenza were 4,051 for pneumonia/influenza category and 15,873 for respiratory/circulatory diseases category per ICD9*.  These exceed rates documented in temperate regions. |
| Wong et al [[4](#_ENREF_4)] | Hong Kong SAR(China) | 1996-2000 | Retrospective review of hospitalization data | All | Laboratory-confirmed influenza was significantly associated with ICD9 category for hospitalization for acute respiratory disease.  Annual rates of excess hospitalizations per 100,000 population for this ICD9 category varied by age, and were highest in children (0-14 years) at 163.3 (95% CI* 135-190) and elderly (≥ 75 years) at 266 (95% CI 198.7-330.2) |
| Yap et al [[5](#_ENREF_5)] | Hong Kong SAR(China) | 1998-2001 | Retrospective population based study from a regional hospital | ≥65 years | Influenza activity was an independent significant factor affecting admission rates for pneumonia, COPD*, and heart failure but not that for asthma. Influenza activity is associated significant excess hospital admissions among elderly. |
| Chiu et al [[6](#_ENREF_6)] | Hong Kong SAR(China) | 1997-1999 | Retrospective population based study from single hospital known for intensive use of laboratory-testing | ≤ 15 years | Very young children had highest rates of hospitalization for influenza:   - < 1 year: 288.2 per 10,000 population - 1-2 years: 209.3 per 10,000 population   These rates exceed those reported from temperate regions such as Australia. |
| Beard et al [[7](#_ENREF_7)] | Australia | 1994-2001 | Retrospective population based study using two methods to estimate hospitalization rates | ≤ 18 years | Hospitalization rates for influenza based on virological surveillance data combining data on influenza and RSV* were 11 times greater than rates of hospitalization based on influenza being the principal discharge diagnosis for children (≤ 2 years). |
| Kwong et al [[8](#_ENREF_8)] | Hong Kong SAR(China) | 2005 (6 months) | Retrospective study in one regional hospital serving 180,000 population | ≤ 18 years | Influenza A accounted for 93.5% of hospitalizations. Children <5 years age comprised 70% of admission. Mean duration of hospitalization was 3 days (range 0.5–12.5 days). |
| Chiu et al [[9](#_ENREF_9)] | Hong Kong SAR(China) | 2003-2006 | Prospective study in two hospitals that serve 72.5% of all general pediatric admissions | <18 years | Children <1 year old had highest hospitalization rate for influenza A in 2004-2005 (103.8 per 10,000). Hospitalization rates varied by virus type and subtype. |
| D’Onise et al [[10](#_ENREF_10)] | Australia | 1996-2006 | Retrospective study of hospital separation data with 649 children admitted for influenza | <5 years | High admission rates for children <1 year old: 151 per 100,000.  For Indigenous children, high rates were observed for children <5 years old: 161.8 per 100,000. Most (81%) children hospitalized did not have underlying illnesses. |
| Mermond et al [[11](#_ENREF_11)] | New Caledonia | 2006-2007 | Prospective study of 137 hospitalized community-acquired pneumonia cases. | All ages | Influenza virus was detected in 4 patients (15% of those tested for influenza). Common coinfections were S. pneumonia with influenza A virus. |
| Drennan et al [[12](#_ENREF_12)] | Australia | 2009 | Surveys to assess critical care unit utilization during the 2009 pandemic. | All ages | A(H1N1)pdm09 cases required approximately 12.4% of the ventilator resources and used 8.1% of total patient bed-days. |
| Shin et al [[13](#_ENREF_13)] | Republic of Korea | 2010 | Retrospective study of 30 critically-ill pediatric patients infected with A(H1N1)pdm09 virus. | <18 years | 14 cases died, where the most common causes of death were encephalopathy and myocarditis. |
| Tomizuka et al [[14](#_ENREF_14)] | Japan | 2010 | Retrospective study of surveillance data for A(H1N1)pdm09 hospitalized patients. | All ages | Hospitalization incidence was 10.0 admissions/100,000 persons.  Attributable risk for medical conditions associated with hospitalization was highest among patients 5–9 and 10–14 years of age (744.7 and 549.6, respectively), whereas the risk was lowest (6.5) among patients 60–69 years of age. |
| Ong et al [[15](#_ENREF_15)] | Malaysia | 2009 | Economic analysis of costs for 609 influenza patients in a teaching hospital. | All ages | Cost of direct healthcare of a A(H1N1)pdm09 infection was USD 510. This was 60% greater than per capita national expenditure on health of USD 318.  Cost was higher if patients had risk factors for severe influenza. |

* ICD9 - International Classification of Diseases version 9 codes, 95% CI – 95% Confidence Interval, RSV - respiratory syncytial virus, COPD – chronic obstructive pulmonary disease

References

1. Verrall A, Norton K, Rooker S, Dee S, Olsen L, et al. (2010) Hospitalizations for pandemic (H1N1) 2009 among Maori and Pacific Islanders, New Zealand. Emerg Infect Dis 16: 100-102.

2. Yu H, Feng L, Peng Z, Feng Z, Shay DK, et al. (2009) Estimates of the impact of a future influenza pandemic in China. Influenza Other Respi Viruses 3: 223-231.

3. Li CK, Choi BC, Wong TW (2006) Influenza-related deaths and hospitalizations in Hong Kong: a subtropical area. Public Health 120: 517-524.

4. Wong CM, Yang L, Chan KP, Leung GM, Chan KH, et al. (2006) Influenza-associated hospitalization in a subtropical city. PLoS Med 3: e121.

5. Yap FH, Ho PL, Lam KF, Chan PK, Cheng YH, et al. (2004) Excess hospital admissions for pneumonia, chronic obstructive pulmonary disease, and heart failure during influenza seasons in Hong Kong. J Med Virol 73: 617-623.

6. Chiu SS, Lau YL, Chan KH, Wong WH, Peiris JS (2002) Influenza-related hospitalizations among children in Hong Kong. N Engl J Med 347: 2097-2103.

7. Beard F, McIntyre P, Gidding H, Watson M (2006) Influenza related hospitalisations in Sydney, New South Wales, Australia. Arch Dis Child 91: 20-25.

8. Kwong KL, Lung D, Wong SN, Que TL, Kwong NS (2009) Influenza-related hospitalisations in children. J Paediatr Child Health 45: 660-664.

9. Chiu SS, Chan KH, Chen H, Young BW, Lim W, et al. (2009) Virologically confirmed population-based burden of hospitalization caused by influenza A and B among children in Hong Kong. Clin Infect Dis 49: 1016-1021.

10. D'Onise K, Raupach JC (2008) The burden of influenza in healthy children in South Australia. Med J Aust 188: 510-513.

11. Mermond S, Berlioz-Arthaud A, Estivals M, Baumann F, Levenes H, et al. (2010) Aetiology of community-acquired pneumonia in hospitalized adult patients in New Caledonia. Trop Med Int Health 15: 1517-1524.

12. Drennan K, Hicks P, Hart G (2010) Impact of pandemic (H1N1) 2009 on Australasian critical care units. Crit Care Resusc 12: 223-229.

13. Shin SY, Kim JH, Kim HS, Kang YA, Lee HG, et al. (2010) Clinical characteristics of Korean pediatric patients critically ill with influenza A (H1N1) virus. Pediatr Pulmonol 45: 1014-1020.

14. Tomizuka T, Takayama Y, Shobayashi T, Fukushima Y, Suzuki Y (2010) Underlying medical conditions and hospitalization for pandemic (H1N1) 2009, Japan. Emerging infectious diseases 16: 1646-1647.

15. Ong MP, Sam IC, Azwa H, Mohd Zakaria IE, Kamarulzaman A, et al. (2010) High direct healthcare costs of patients hospitalised with pandemic (H1N1) 2009 influenza in Malaysia. The Journal of infection 61: 440-442.
