# Supplementary material for: A Review of the Evidence to Support Influenza Vaccine Introduction in Countries and Areas of WHO's Western Pacific Region
Source: PLoS One. 2013 Jul 16;8(7):e70003. doi: 10.1371/journal.pone.0070003 (PMC3713047; doi:10.1371/journal.pone.0070003)
Supplement: Table S3 — Studies reporting on influenza-associated mortality. (DOCX) [file pone.0070003.s003.docx]

Supplementary Table 3: Studies reporting on influenza-associated mortality

| **Author** | **Country** | **Year(s)** | **Study description** | **Age group** | **Key findings** |
| --- | --- | --- | --- | --- | --- |
| Lee et al [[1](#_ENREF_1)] | Singapore | 1950-2000 | Retrospective population based study based on death statistics and virus surveillance data | All | Excess mortality could be observed in almost all periods with recorded influenza outbreaks but did not always exceed the 95% CI* of the baseline mortality rate. Post 1972, influenza epidemics were the likely cause of most excess mortality periods. |
| Li et al [[2](#_ENREF_2)] | Hong Kong SAR(China) | 1999-2000 | Retrospective population based study using national mortality data, and influenza surveillance system data | All | Annual mean excess numbers of deaths related to influenza were estimated to be 613 for pneumonia/influenza and 2,302 for respiratory/circulatory diseases. The crude influenza-related mortality rates in Hong Kong exceeded those documented in temperate regions. |
| Chow et al [[3](#_ENREF_3)] | Singapore | 1996-2003 | Retrospective population based study | All | Influenza was associated with an annual death rate from all causes. An estimated 6.5% of underlying pneumonia and influenza deaths were attributable to influenza. Results are comparable with the United States and Hong Kong. Proportion of influenza-associated deaths was 11.3 times higher in persons age >65 years than in the general population. |
| He et al [[4](#_ENREF_4)] | China | 1999-2000 | Retrospective cohort study of 169,871 adults | ≥40 years | The mortality from all causes was 1480.1 per 100,000 person-years among men and 1190.2 per 100,000 person-years among women. Fourth leading cause of death in women was pneumonia/influenza (45.9 per 100,000 person-years). |
| Wong et al [[5](#_ENREF_5)] | Hong Kong SAR(China) | 1996-1999 | Retrospective population based study using national mortality data |  | Although respiratory diseases accounted for the majority of influenza-related deaths, influenza also contributed to 13.8% (95% CI*, 4.8%- 22.7%) and 5.3% (95% CI, 1.2%-9.3%) of deaths related to ischemic heart disease. Rates comparable to temperate countries. |
| Yu et al [[6](#_ENREF_6)] | China | 2009 | Monte Carlo simulation model | All | Assuming 1968 pandemic strain severity, a pandemic would lead to 460,000-700,000 deaths. |
| Reichert et al [[7](#_ENREF_7)] | Japan | 1949-1998 | Retrospective population based study based on death statistics data and census data on rates of vaccination | All | Vaccinating schoolchildren against influenza prevented 37,000 to 49,000 deaths per year, or about 1 death for every 420 children vaccinated. The program likely reduced elderly influenza-related mortality. |
| Sugaya et al [[8](#_ENREF_8)] | Japan | 1972-2003 | Retrospective study of mortality data | 1-4 years | Excess deaths were estimated to be 783 in the 11 winter seasons when vaccination of schoolchildren was discontinued (1990-2000). No winter peaks in deaths were seen after 2000 when vaccination of young (1-4 year old) children and use of antivirals increased. |
| Yang et al [[9](#_ENREF_9)] | Hong Kong SAR(China) | 1996-2002 | Retrospective study using Poisson regression models fitted to weekly numbers of mortality | All | Influenza-related mortality was higher in winter and late spring/early summer than other seasons. The two-peak pattern was found for cardio-respiratory disease and pneumonia/influenza, COPD, cerebrovascular diseases and ischemic heart disease as well as for all-cause deaths. |

* 95% CI – 95% Confidence Interval, COPD – chronic obstructive pulmonary disease

References

1. Lee VJ, Yap J, Ong JB, Chan KP, Lin RT, et al. (2009) Influenza excess mortality from 1950-2000 in tropical Singapore. PLoS One 4: e8096.

2. Li CK, Choi BC, Wong TW (2006) Influenza-related deaths and hospitalizations in Hong Kong: a subtropical area. Public Health 120: 517-524.

3. Chow A, Ma S, Ling AE, Chew SK (2006) Influenza-associated deaths in tropical Singapore. Emerg Infect Dis 12: 114-121.

4. He J, Gu D, Wu X, Reynolds K, Duan X, et al. (2005) Major causes of death among men and women in China. N Engl J Med 353: 1124-1134.

5. Wong CM, Chan KP, Hedley AJ, Peiris JS (2004) Influenza-associated mortality in Hong Kong. Clin Infect Dis 39: 1611-1617.

6. Yu H, Feng L, Peng Z, Feng Z, Shay DK, et al. (2009) Estimates of the impact of a future influenza pandemic in China. Influenza Other Respi Viruses 3: 223-231.

7. Reichert TA, Sugaya N, Fedson DS, Glezen P, Simonsen L, et al. (2001) The Japanese experience with vaccinating schoolchildren against influenza. N Engl J Med 344: 889-896.

8. Sugaya N, Takeuchi Y (2005) Mass vaccination of schoolchildren against influenza and its impact on the influenza-associated mortality rate among children in Japan. Clin Infect Dis 41: 939-947.

9. Yang L, Wong CM, Chan KP, Chau PY, Ou CQ, et al. (2009) Seasonal effects of influenza on mortality in a subtropical city. BMC Infect Dis 9: 133.
